# Multiple-stressor effects in an apex predator: combined influence of pollutants and sea ice decline on lipid metabolism in polar bears

**DOI:** 10.1038/s41598-017-16820-5

**Published:** 2017-11-28

**Authors:** Sabrina Tartu, Roger Lille-Langøy, Trond R. Størseth, Sophie Bourgeon, Anders Brunsvik, Jon Aars, Anders Goksøyr, Bjørn Munro Jenssen, Anuschka Polder, Gregory W. Thiemann, Vidar Torget, Heli Routti

**Affiliations:** 1 Norwegian Polar Institute, Fram Centre, NO-9296 Tromsø, Norway; 2 University of Bergen, Department of Biology, NO-5020 Bergen, Norway; 3 SINTEF, Trondheim, Norway; 4 UiT-The Arctic University of Norway, Department of Arctic and Marine Biology, NO- 9019 Tromsø, Norway; 5 Norwegian University of Science and Technology, Department of Biology, NO-7491 Trondheim, Norway; 6 Norwegian University of Life Science, Campus Adamstua, NO- 0033 Oslo, Norway; 7 York University, Faculty of Environmental Studies, Toronto, ON M3J 1P3 Canada

## Abstract

There is growing evidence from experimental and human epidemiological studies that many pollutants can disrupt lipid metabolism. In Arctic wildlife, the occurrence of such compounds could have serious consequences for seasonal feeders. We set out to study whether organohalogenated compounds (OHCs) could cause disruption of energy metabolism in female polar bears (*Ursus maritimus*) from Svalbard, Norway (n = 112). We analyzed biomarkers of energy metabolism including the abundance profiles of nine lipid-related genes, fatty acid (FA) synthesis and elongation indices in adipose tissue, and concentrations of lipid-related variables in plasma (cholesterol, high-density lipoprotein, triglycerides). Furthermore, the plasma metabolome and lipidome were characterized by low molecular weight metabolites and lipid fingerprinting, respectively. Polychlorinated biphenyls, chlordanes, brominated diphenyl ethers and perfluoroalkyl substances were significantly related to biomarkers involved in lipid accumulation, FA metabolism, insulin utilization, and cholesterol homeostasis. Moreover, the effects of pollutants were measurable at the metabolome and lipidome levels. Our results indicate that several OHCs affect lipid biosynthesis and catabolism in female polar bears. Furthermore, these effects were more pronounced when combined with reduced sea ice extent and thickness, suggesting that climate-driven sea ice decline and OHCs have synergistic negative effects on polar bears.

## Introduction

Since many chemicals are lipid-soluble, it is relevant to investigate whether their concurrent and continuous presence in adipose tissues may be harmful^1^. A major role of adipose tissue is to store ingested energy in the form of triglycerides during periods of energy excess and make it available during periods of energy deprivation^2^. Recently it has been recognized that adipose tissue is an endocrine organ involved in functions such as immune response, inflammation, reproduction and metabolism^3,4^. In mammals, the regulation of lipid metabolism is the result of the concomitant action of the central nervous system and several organs such as the liver, muscles and adipose tissue, via hormonal messengers. In white adipocytes, fatty acid (FA) transport, synthesis, uptake and lipid hydrolysis are controlled by a set of genes mostly regulated by “the master regulator of adipogenesis”: the nuclear receptor peroxisome-proliferator activated receptor gamma (PPARG)^5^. Several factors interact with PPARG to facilitate lipid metabolism (e.g. lipid synthesis, storage and hydrolysis). These include, for example, the PPARG-coactivator-1 (PGC1), which increases the expression of genes encoding respiratory chain proteins, enzymes of FA oxidation, and causing white adipocytes to acquire features of brown adipocytes^6^. Another factor interacting with PPARG is the sterol regulatory element-binding protein 1 (SREBP1)^7^, which leads to increased FA synthesis and their storage as triglycerides in adipocytes and peripheral tissues^8–10^. *Cluster of differentiation 36* (*CD36*) and *FA binding protein 4* (*FABP4*) are PPARG target genes whose gene products are involved in FA uptake and transport^10^. Moreover, the FA synthase (FASN) induces FA synthesis and produces ligands to PPARG^10,11^. The breakdown of triglycerides to free FAs (FFAs) and diacylglycerol occurs via the action of the patatin-like phospholipase domain-containing protein 2 (PNPLA2), another PPARG target gene product, and the second hydrolysis step (break down of diacylglycerol to monoacylglycerol and FFAs) is catalyzed by lipase E (LIPE, also known as hormone sensitive lipase) and other lipases^12^. Adiponectin (ADIPOQ), a protein hormone regulated by PPARG, sensitizes insulin and stimulates energy metabolism in tissues^13,14^.

Several experimental and epidemiological studies on mammals have indicated that organohalogenated compounds (OHCs) such as polychlorinated biphenyls (PCBs), organochlorine pesticides (OCPs), polybrominated diphenyl ethers (PBDEs) and poly- and perfluoroalkyl substances (PFASs) affect lipid metabolism^15–19^. Arctic top predators, such as polar bears (*Ursus maritimus*), are among the most polluted species in the world^20^ and have evolved large body lipid stores in response to seasonal variation in prey availability and cold temperatures^21^. Polar bears have a unique metabolism that enables them to deal with a lipid-rich diet and to efficiently accumulate adipose tissue during periods of food abundance^22,23^. Within a few months (generally from April to July), polar bears feed actively and can accumulate up to 50% of their body mass as lipids, through consumption of seal blubber during the ringed seal (*Pusa hispida*) pupping and moulting periods^24^. These energy stores are metabolized during periods of food scarcity or reproductive fasting. For instance, polar bears frequently fast in autumn if stranded in areas without access to sea ice, and prey encounter is thought to be low in winter^25^. Female polar bears can sustain a 4–8 month fasting period (late autumn/early winter to the following spring) during denning, associated with parturition and lactation^26^. The reproductive success of female polar bears thus directly depends on their lipid stores^24^.

There is a lack of information on how pollutants may affect energy metabolism in free-ranging polar bears. An appropriate response to seasonal feeding is to accumulate triglycerides in adipose tissue when food is available and release this energy in the form of FAs and glycerol in times of food scarcity. Routti *et al*. (2016) recently showed that lipophilic pollutants (e.g. PCBs, OCPs and brominated flame retardants: BFRs) stored in polar bear adipose tissue modulate the polar bear PPARG function and the differentiation of polar bear stem cells into adipocytes^27^. Furthermore, pollutants are suspected to impair thyroid hormone concentrations in polar bears^28–32^ and thyroid hormones are known to be involved in the synthesis, mobilization and degradation of lipids^33,34^. Improved knowledge of the effects of pollutants on energy metabolism is crucial to understanding how polar bears will respond to ongoing habitat loss associated with global warming^35^. Sea ice decline has been documented to result in longer periods of fasting, reduced foraging opportunities, lower body condition, decreased access to denning areas and lower survival of cubs^36–38^. Furthermore, declining sea ice has contributed to increased concentrations of lipophilic pollutants in polar bears due to a decrease in body condition^39^. Impaired lipid metabolism caused by pollutant exposure may have significant consequences on polar bear physiology and reproduction^35^. Within the circumpolar habitat of polar bears, the Barents Sea has been experiencing the fastest loss of sea ice extent over the past decades^40^, and polar bears from that area are among the most polluted Arctic wildlife populations with respect to OHCs^20,41–45^. The combination of these two factors may put Barents Sea polar bears at especially high risk to multi-stress effects by pollutants and climate change.

The main goal of the present study was to investigate the effects of OHC exposure on energy metabolism in Barents Sea polar bears at multiple levels. In addition, we described how energy biomarkers varied according to nutritional state (feeding or fasting individuals) and we investigated whether sea ice conditions interact with the effects of OHCs on energy metabolism in polar bears. Specifically, we investigated whether the response of energy metabolism to pollutant exposure differed when sea ice was less extended and bears were under nutritional stress compared to more “seasonally adequate” sea ice conditions^39^. The biomarkers of energy metabolism included targeted analyses (i.e. gene transcription and FA metabolism in adipose tissue, and concentrations of lipid related variables in plasma), and non-targeted end-points such as plasma metabolome and lipidome, which are considered as final downstream products of gene transcription.

## Results

### Targeted biomarkers of energy metabolism

We assumed that most females captured in April would be in a feeding state whereas most of those captured in September would be in a fasting state. In female polar bears, which were in a feeding state, we measured higher expressions of *PPARG*, *PGC1* and *FASN* transcript levels **(**Table S1) in adipose tissue as compared to fasting bears. Fasting females had increased *de novo* FA synthesis, decreased FA elongation index and higher plasma concentrations of triglycerides (Table S1). Several variables related to lipid metabolism remained unchanged between the two metabolic states (feeding or fasting), including *PNPLA2*, *LIPE*, *ADIPOQ*, *CD36* and *FABP4* transcript levels and concentrations of cholesterol and high-density lipoprotein (HDL) (Table S1).

**Figure 1:**
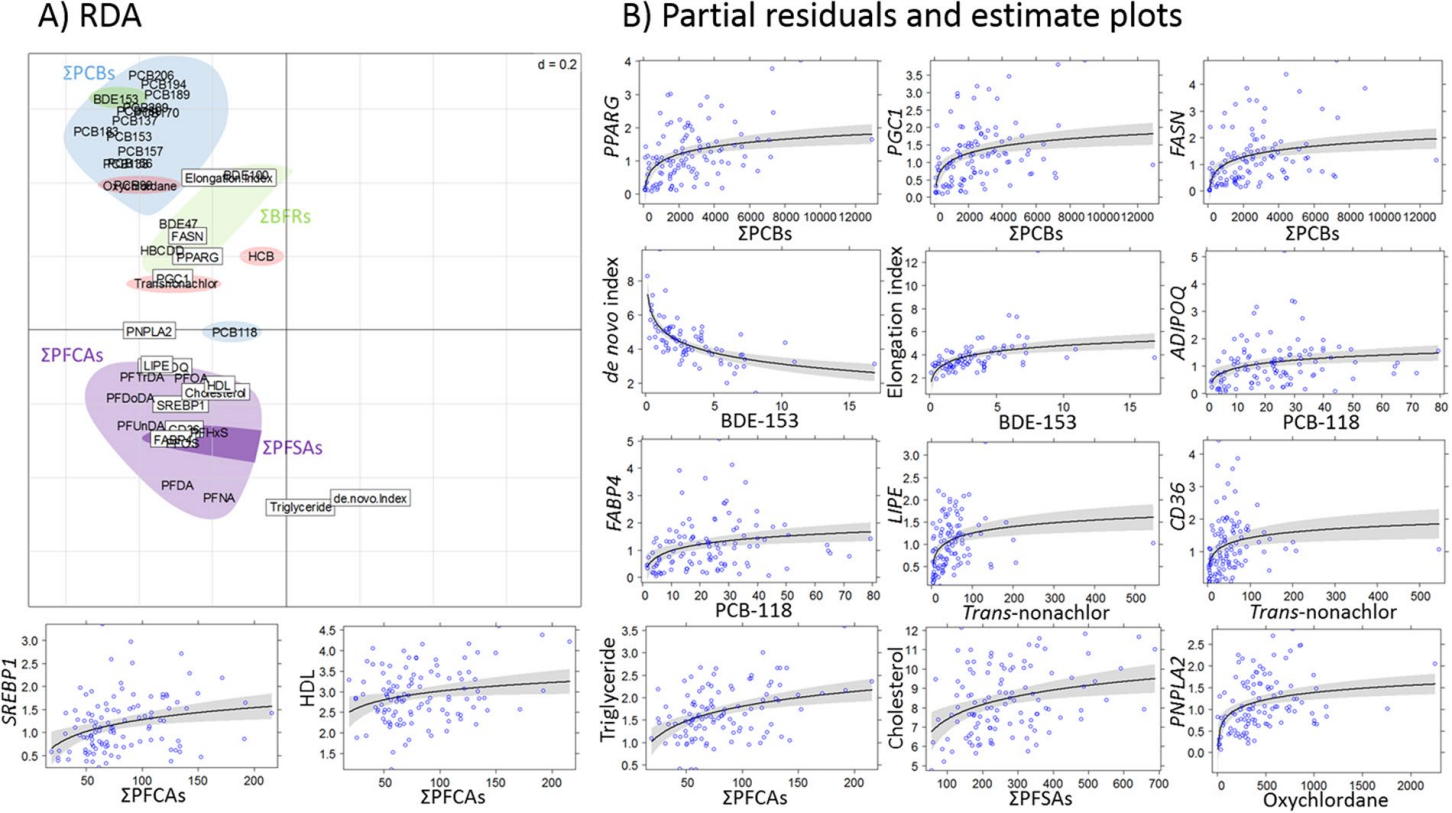
Relationships between biomarkers of energy metabolism and pollutants. (**A**) Redundancy analysis (RDA) loading plot (n = 80) and (**B**) partial residuals and estimate plots obtained from mixed models (n = 111). Plasma and fat samples are from Svalbard female polar bears captured during spring (April) and autumn (September) 2012 and 2013. For the RDA (**A**), boxed labels are response variables and unboxed labels are predictors. (**B**) Dots are the partial residuals, the solid line is the parameter estimate and the grey area represents its 95% confidence interval derived from mixed models. Pollutant concentrations are in ng/g wet weight, transcript levels are in arbitrary units, lipid parameters in mmol/L.

The pollutants included in the redundancy analyses (RDA) explained 62% of the variation of the biomarkers of energy metabolism, and the RDA model was highly significant based on Monte-Carlo test (1000 replicates, RV coefficient = 0.49, p < 0.001). According to their proximity on the RDA plot, some pollutants were summed to reduce the number of predictors used in generalized linear mixed models (GLMMs), resulting in nine pollutant predictors (Fig. 1A). Oxychlordane, *trans*-nonachlor, hexachlorobenzene (HCB), PCB-118 and BDE-153 were considered individually, whereas the remaining compounds were summed as follows: Σ_13_PCBs (PCB-99, -137, -138, -153, -156, -157, -170, -180, -183, -189, -194, -206 and -209), Σ_3_ brominated flame retardants (BFRs: BDE-47, -100, hexabromocyclododecane), Σ_6_ perfluoroalkyl carboxylates (PFCAs with carbon chain length of 8 to 13) and Σ_2_ perfluoroalkyl sulfonates (PFSAs with 6 and 8 carbons). Pollutant concentrations (mean ng/g wet weight ± SD) in polar bear adipose tissue were 2607 ± 2137 for ∑_13_PCBs, 22.8 ± 15.9 for PCB-118, 62.2 ± 75.9 for HCB, 460 ± 349 for oxychlordane, 53.0 ± 62.5 for *trans*-nonachlor, 22.1 ± 13.8 for ∑_3_BFRs and 3.37 ± 3.08 for BDE-153. Plasma concentrations (ng/g wet weight) of ∑_2_PFSAs and ∑_6_PFCAs were 264 ± 130 and 81.7 ± 38.0, respectively.

Relationships between biomarkers of energy metabolism and pollutants were consistent between RDA and model averaging estimates (Fig. 1A,B, Table 1). To identify the strongest predictors, we focused on the top models explaining the variation in biomarkers of energy metabolism (ΔAICc < 2). The three most competitive models and model averaged estimates for these models are given in Table 1. Overall, three major groups of pollutants explained most of the variation of biomarkers of energy metabolism. Specifically, ΣPCBs, BDE-153 and oxychlordane were the strongest predictors for *PPARG*, *PGC1*, *FASN*, *PNPLA2* transcript levels and FA indexes (*de novo* synthesis and elongation). *Trans*-nonachlor and PCB-118 were the strongest predictors for *LIPE*, *ADIPOQ*, *CD36* and *FABP4* transcript levels. ΣPFCAs and ΣPFSAs were among the strongest predictors for *SREBP1* transcript levels, and, concentrations of cholesterol, HDL and triglycerides (Table 1). Generally, pollutants predicted increases of biomarkers of energy metabolism, except for BDE-153, which predicted a decrease of *de novo* FA synthesis (Fig. 1B, Table 1).

**Table 1:** Relationships between biomarkers of energy metabolism and pollutant concentrations in female polar bears adipose tissue and plasma captured in Svalbard (2012–2013).

Response variable	Predictor	Three most competitive models					Conditional average estimates and 95% CI	
		df	log Likelihood	AICc	ΔAICc	weight	Intercept	Predictor
*PPARG*	**ΣPCBs**	**5**	**−122.16**	**254.89**	**0**	**0.69**	**−**0.69 [**−**2.68; 1.29]	**0.32 [0.18; 0.47]**
	BDE-153	5	**−**123.22	257.02	2.13	0.24		**0.33 [0.17; 0.48]**
	Oxychlordane	5	**−**124.61	259.8	4.92	0.06		**0.30 [0.15; 0.45]**
*PGC1*	**ΣPCBs**	**5**	**−127.19**	**264.96**	**0**	**0.52**	**−**0.38 [**−**2.21; 1.45]	**0.30 [0.15; 0.45]**
	**BDE-153**	**5**	**−127.96**	**266.5**	**1.54**	**0.24**		**0.30 [0.14; 0.47]**
	Oxychlordane	5	**−**128.31	267.19	2.23	0.17		**0.29 [0.13; 0.45]**
*FASN*	**ΣPCBs**	**5**	**−151.86**	**314.29**	**0**	**0.52**	**−**0.62 [**−**2.99; 1.75]	**0.37 [0.18; 0.55]**
	**BDE-153**	**5**	**−152.34**	**315.25**	**0.96**	**0.32**		**0.38 [0.19; 0.58]**
	Oxychlordane	5	**−**153.07	316.72	2.43	0.15		**0.35 [0.16; 0.55]**
*PNPLA2*	**Oxychlordane**	**5**	**−93.65**	**197.87**	**0**	**0.58**	**−**0.21 [**−**1.15; 0.74]	**0.24 [0.12; 0.36]**
	**ΣPCBs**	**5**	**−94.51**	**199.6**	**1.73**	**0.25**		**0.22 [0.11; 0.34]**
	*Trans-*nonachlor	5	**−**95.45	201.47	3.6	0.1		**0.21 [0.09; 0.33]**
*LIPE*	***Trans-*** **nonachlor**	**5**	**−85.14**	**180.86**	**0**	**0.84**	0.29 [**−**0.14; 0.73]	**0.21 [0.10; 0.31]**
	PCB-118	5	**−**87.39	185.36	4.5	0.09		**0.20 [0.08; 0.33]**
	Oxychlordane	5	**−**88.38	187.34	6.48	0.03		**0.17 [0.06; 0.28]**
*ADIPOQ*	**PCB-118**	**5**	**−121.6**	**253.78**	**0**	**0.48**	0.25 [**−**0.51; 1.02]	**0.26 [0.09; 0.44]**
	***Trans-*** **nonachlor**	**5**	**−122.21**	**254.98**	**1.21**	**0.26**		**0.22 [0.07; 0.36]**
	Oxychlordane	5	**−**123.1	256.76	2.99	0.11		**0.20 [0.05; 0.36]**
*CD36*	***Trans-*** **nonachlor**	**5**	**−133.63**	**277.83**	**0**	**0.5**	0.28 [**−**0.42; 0.98]	**0.25 [0.09; 0.41]**
	**PCB-118**	**5**	**−133.92**	**278.42**	**0.59**	**0.37**		**0.29 [0.09; 0.48]**
	Oxychlordane	5	**−**136.26	283.1	5.27	0.04		**0.18 [0.01; 0.35]**
*FABP4*	**PCB-118**	**5**	**−144.18**	**298.92**	**0**	**0.52**	0.17 [**−**0.69; 1.04]	**0.33 [0.12; 0.55]**
	***Trans-*** **nonachlor**	**5**	**−144.57**	**299.72**	**0.79**	**0.35**		**0.27 [0.09; 0.45]**
	ΣPFCAs	5	**−**146.84	304.26	5.34	0.04		**0.45 [0.04; 0.86]**
*SREBP1*	**ΣPFCAs**	**5**	**−92.8**	**196.17**	**0**	**0.44**	0.11 [**−**1.28; 1.50]	**0.37 [0.11; 0.64]**
	***Trans-*** **nonachlor**	**5**	**−93.19**	**196.96**	**0.79**	**0.29**		**0.15 [0.04; 0.27]**
	ΣPFSAs	5	**−**94.55	199.68	3.51	0.08		**0.27 [0.02; 0.52]**
*de novo* synthesis index	**BDE-153**	**5**	**−111.15**	**233.1**	**0**	**0.99**	**5.35 [4.44; 6.26]**	**−0.96 [−1.27; −0.65]**
	ΣPCBs	5	**−**116.38	243.55	10.45	0.01		**−0.89 [−1.24; −0.53]**
	Oxychlordane	5	**−**123.81	258.41	25.31	0		**−0.74 [−1.16; −0.31]**
Elongation index	**BDE-153**	**5**	**−135**	**280.78**	**0**	**0.98**	**3.04 [1.84; 4.23]**	**0.73 [0.32; 1.14]**
	ΣPCBs	5	**−**138.86	288.5	7.72	0.02		**0.58 [0.12; 1.04]**
	Oxychlordane	5	**−**142.02	294.83	14.05	0		0.37 [**−**0.15; 0.9]
Cholesterols	**ΣPFSAs**	**5**	**−205.21**	**420.99**	**0**	**0.63**	3.27 [**−**1.17; 7.71]	**1.08 [0.40; 1.77]**
	**ΣPFCAs**	**5**	**−206.04**	**422.66**	**1.67**	**0.27**		**1.08 [0.35; 1.82]**
	BDE-153	5	**−**207.88	426.33	5.34	0.04		**0.38 [0.04; 0.73]**
HDL	**BDE-153**	**5**	**−105.53**	**221.64**	**0**	**0.33**	**2.32 [1.02; 3.61]**	**0.15 [0.02; 0.29]**
	**ΣPFCAs**	**5**	**−105.99**	**222.56**	**0.91**	**0.21**		**0.30 [0.02; 0.59]**
	ΣPFSAs	5	**−**106.63	223.84	2.2	0.11		0.24 [**−**0.02; 0.50]
Triglycerides	**ΣPFCAs**	**5**	**−84.58**	**179.74**	**0**	**0.84**	0 [**−**1.99; 1.98]	**0.47 [0.24; 0.70]**
	BDE-153	5	**−**86.83	184.23	4.49	0.09		**−0.19 [−0.31; −0.08]**
	ΣPCBs	5	**−**87.73	186.04	6.3	0.04		**−0.17 [−0.28; −0.06]**
Glucose	Null model	4	**−**284.64	577.67	0	0.17	2.58 [**−**2.17; 0.7]	0.39 [**−**2.36; 0.77]
	PCB-118	5	**−**284.03	578.64	0.97	0.11		**−**0.73 [**−**0.34; 0.98]
	ΣPFSAs	5	**−**284.04	578.66	0.99	0.11		**−**0.79 [**−**0.33; 0.89]
Lactate	**ΣPFCAs**	**5**	**38.71**	**−66.84**	**0**	**0.44**	0.41 [**−**0.03; 0.86]	**−0.084 [−0.164; −0.004]**
	ΣPFSAs	5	37.47	**−**64.37	2.47	0.13		**−**0.06 [**−**0.13; 0.02]
	Null model	4	36.22	**−**64.05	2.78	0.11		0.03 [**−**0.02; 0.07]

The three most competitive models including the best predictor (ΔAICc = 0), predictors that received strong support (ΔAICc ≤ 2), conditional averaged estimates and 95% confidence intervals derived from mixed models are given. Bold values represent significant relationships, shaded rows represent the variables and relationships with ΔAICc < 2.

### Non-targeted biomarkers of energy metabolism: Metabolome and lipidome

**Figure 2:**
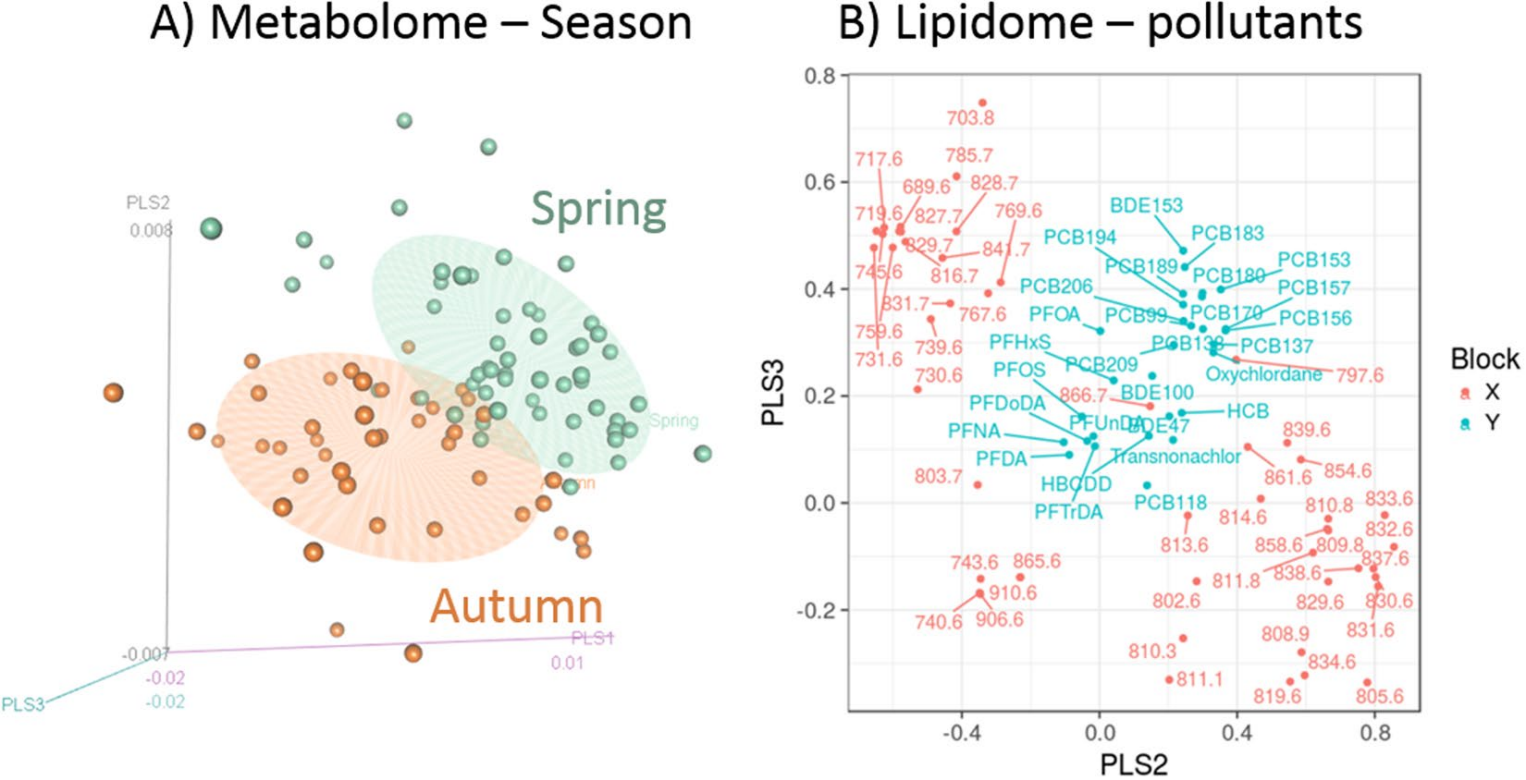
Polar bear metabolome and lipidome. (**A**) Three dimension representation of female polar bears metabolome according to season (n = 111) characterized by low molecular weight metabolites. The three axes are obtained from partial least squares scores. (**B**) Female polar bear lipidome in relation pollutants. The lipidome is characterized by lipid fingerprinting determined in female polar bear plasma (n = 101). The figure represents lipids exact mass (m/z) in pink and pollutants in blue. Bears were sampled at Svalbard, Norway during spring (April: green dots in A) and autumn (September: orange dots in A) 2012 and 2013.

The partial least square (PLS) scores for polar bear metabolome significantly clustered according to season (MANOVA, Pillai trace test, F = 31.7, p < 0.001, Fig. 2A). The loadings (Figure S2) indicated that glucose and lactate were the main metabolites driving seasonal metabolome segregation with higher concentrations of glucose and lactate in feeding (April) compared to fasting (September) females (see Table S1 for estimates and 95% CI according to season). Other metabolites that had less influence on the metabolome segregation were not quantifiable due to misalignment of peaks, which may have resulted from the presence of plastic polymers in the sampling tubes or heparin in the plasma samples^46,47^. For the lipidome, the PLS scores also clustered according to season (MANOVA, Pillai trace test, F = 53.09, p < 0.001).

Plasma ΣPFCA concentrations predicted a decrease of lactate concentrations, whereas OHCs did not influence glucose concentrations in plasma (Table 1). A PLS discriminant analysis correlation plot on the lipidome and pollutants clustered pollutants mainly into three groups, one containing PCBs, oxychlordane and BFRs, another one containing PFASs (Fig. 2B), and the third cluster containing *trans*-nonachlor, PCB-118 and HCB. The third cluster slightly correlated to a lipid with a mass of 866.7 m/z for which it was not possible to assign the mass of any specific lipid due to low abundance in Fourier transform ion cyclotron resonance (FTI-CR). A lipid with a mass of 797.6 m/z was found to correlate with the PCBs, oxychlordane and BFRs cluster using the time-of-flight mass-spectrometry data, which was assigned as the isotope peak of 796.6 using the FT-ICR coupled to mass spectrometry (glycerophosphocholine 37:3).

### Declining sea ice exacerbates the effects of pollutants on polar bear energy metabolism

During the three periods where availability to sea ice was highest (i.e. April and September 2012 and September 2013), the metabolic responses of female polar bears were not different between females with high versus low levels of pollution: a maximum of two biomarkers of energy metabolism out of 16, had significantly different responses according to pollutant level (Table 2). In contrast, among bears sampled when access to sea ice was really low (April 2013), the response of seven energy metabolism biomarkers were higher in the more polluted females compared to the less polluted ones (Table 2). In addition, when we consider the biomarkers with responses close to significance (0.05 < p < 0.10), the response to pollutants of one more biomarker presented differences between the high and low pollution groups during periods with good access to sea ice, whereas during the period with low access to sea ice, this was observed in four more biomarkers (Table 2).

**Table 2:** Combined effects of sea ice and pollutants on energy metabolism.

		Pollutant concentrations: High *vs* low			
		April 2012, n = 33	September 2012, n = 24	April 2013 (Stressful), n = 29	September 2013, n = 26
Transcript levels	*PPARG* ^a^	High = Low	High = Low	High > Low*	High = Low
	*PGC1* ^a^	High = Low	High = Low	High > Low*	High = Low
	*FASN* ^a^	High = Low	High > Low•	High > Low•	High = Low
	*PNPLA2* ^a^	High = Low	High = Low	High > Low*	High = Low
	*LIPE* ^b^	High = Low	High = Low	High > Low*	High = Low
	*ADIPO*Q^b^	High > Low•	High = Low	High > Low•	High = Low
	*CD36* ^b^	High = Low	High = Low	High > Low*	High = Low
	*FABP4* ^b^	High = Low	High = Low	High = Low	High = Low
	*SREBP1* ^c^	High > Low*	High > Low*	High = Low	High = Low
FA indexes	*de novo* synthesis^e^	High < Low*	High = Low	High < Low•	High < Low*
	Elongation^e^	High = Low	High = Low	High > Low•	High = Low
Plasma parameters	Cholesterol^d^	High = Low	High = Low	High > Low*	High = Low
	HDL^c^	High = Low	High = Low	High > Low*	High = Low
	Triglycerides^c^	High = Low	High > Low*	High = Low	High = Low
	Lactate^c^	High = Low	High = Low	High = Low	High < Low*

Energy metabolism response to High or Low pollutant concentrations during stressful (spring 2013) and non-stressful (spring and autumn 2012, autumn 2013) sampling periods. We used spring 2013 as a reference “stressful period”. For each group of pollutant (best predictor according to AICc), the data set was split into two classes separated by the median pollutant concentration, resulting in one high and one low polluted group for each individual or group of pollutants. “High” refers to more polluted females with pollutant and “Low” to less polluted females. Pollutants used for high - low classification: ^a^Σ(_13_PCBs, oxychlordane), ^b^Σ(PCB-118, *trans*-nonachlor), ^c^Σ_6_PFCA, ^d^Σ_2_PFSA, ^e^BDE-153. *p < 0.05, ^•^p < 0.10.

## Discussion

Most biomarkers of energy metabolism in female polar bears varied in a predictable way between seasons. During their feeding state in April, the metabolic response of female polar bears resulted in higher expressions of *PPARG*, *PGC1* and *FASN* and higher concentrations of lactate and glucose compared to fasting September females. These biomarkers are involved in gluconeogenesis and FA storage, which would logically occur during a feeding state^10^. *SREBP1* transcript levels, *de novo* FA synthesis and triglyceride concentrations were higher whereas FA elongation index was lower in fasting than in feeding female polar bears. The concomitant action of SREBP1 and PPARG lead to increased synthesis of FA which are stored as triglycerides in adipocytes and peripheral tissues^7,8^. Therefore, an up-regulation of *SREBP1* transcript levels, *de novo* FA synthesis and triglyceride concentrations in fasting bears is surprising. We would have expected this pattern in feeding females (April) when *PPARG* transcript levels were also upregulated and when polar bears are supposed to store lipids. Several parameters related to lipid metabolism remained unchanged between feeding and fasting females. This could occur because polar bears express some of the measured biomarkers regardless of the metabolic state and have a flexible metabolism that enables them to shift from a feeding to a fasting state regardless the season^48^.

The absence or occurrence of seasonal variations in biomarkers of energy metabolism could also result from pollutant exposure. ΣPCBs, BDE-153 and oxychlordane in polar bear adipose tissue predicted an increase of *PPARG*, *PGC1*, *FASN* and *PNPLA2* transcript levels. These four genes are involved in the accumulation of triglycerides via glucose and FFA utilization^6,49,50^. Accordingly, a recent *in vitro* study by Routti *et al*. (2016) showed that pollutants extracted from polar bear tissue increased accumulation of triglycerides in polar bear adipose tissue-derived stem cells during adipogenesis. In contrast, a synthetic mixture of pollutants containing only PCBs and organochlorine pesticides, did not affect triglyceride accumulation^27^. The underlying mechanisms for positive relationships between *PPARG* transcript levels and ΣPCBs, BDE-153 and oxychlordane exposure are likely related to other processes than direct activation of PPARG by the pollutants. In the *in vitro* study the most abundant POPs in polar bear adipose tissue (PCB-153 and oxychlordane) and a mixture of PCBs, chlorinated pesticides and PBDEs reflecting concentrations in polar bear adipose tissue, antagonized polar bear PPARG^27^. However, the *in vitro* study tested whether pollutants would activate a fusion protein of the yeast GAL4-DBD and the PPARG ligand-binding domain^27^, but *in vivo* PPARG forms a permissive heterodimer with retinoid X receptor (RXR), by which RXR ligands can also activate the PPARG/RXR dimer^51,52^.

T*rans*-nonachlor and the dioxin-like (coplanar) PCB-118 predicted an increase of *LIPE*, *ADIPOQ*, *CD36* and *FABP4* transcript levels. The resulting proteins of these four genes affect both the activity and transcript level of each other. For instance, in mice adipocytes, FABP4 and ADIPOQ, respectively, increase and suppress LIPE catalytic activity^14,53^. Moreover ADIPOQ up-regulates CD36 in human macrophage foam cell formation^54^. The observed relationships are likely to stem from effects involving the aryl hydrocarbon receptor (AhR)^55^. Dioxin-like PCBs (e.g. PCB-118) are *AhR* agonists^56^ and long-term activation of AhR induces hepatic accumulation of triglycerides in transgenic mice, likely owing to the combined up-regulation of CD36, FA transport proteins and suppression of FA oxidation^57^. Similarly, *trans*-nonachlor could induce the AhR pathway up-stream or down-stream. Indeed, *trans*-nonachlor gavage in rats induced liver cell hypertrophy and increased hepatic total lipids, triglycerides and phospholipids^58,59^. These pathways have been extensively studied in the liver, but they likely also occur in adipose tissue, since *AhR* is expressed in white adipose tissue of several mammals^60^. Therefore, we may assume that PCB-118 and *trans*-nonachlor could disrupt FA transport, oxidation and insulin utilization *via* the AhR.

Finally, PFASs were related to *SREBP1* transcript levels and biomarkers of energy metabolism in plasma. This association could involve a disruption of the hypothalamic-pituitary-thyroid (HPT) axis. As previously mentioned, PFASs predicted decreased concentrations of free triiodothyronine (T3) in the present female polar bears^31^. In humans, and in murine and primate models, studies have reported negative associations between thyroid hormones and lipid related plasma parameters such as cholesterol, triglycerides and HDL^61–63^. These studies suggest that a disruption of thyroid hormones leads to disrupted levels of plasma lipids. SREBP1 is likely an intermediate for these relationships. T3 represses *SREBP1* expression in human adipocytes, therefore lower T3 concentrations result in increased *SREBP1* expression^64^. Further, SREBP1 is involved in the regulation of cholesterol homeostasis and gluconeogenesis^65,66^. In addition, in human dermal fibroblasts, T3 enhances the production of lactate^67^. Thus, the PFAS-associated decrease of thyroid hormones could account for the decrease of lactate we observed in Svalbard polar bears.

**Figure 3:**
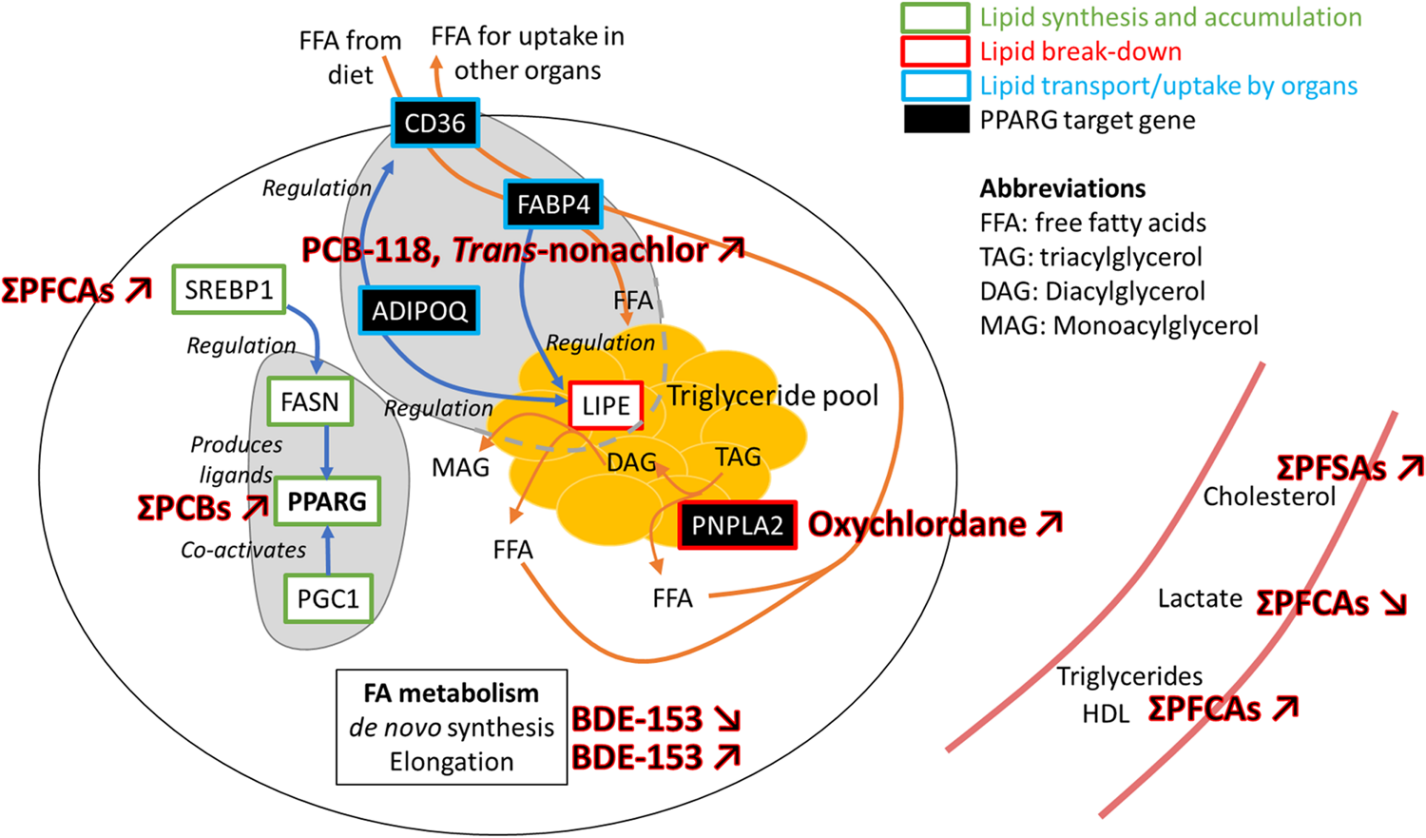
Schematized functions of the genes of interest and summary of the relationships between pollutants and biomarkers of energy metabolism in (**A**) a white adipocyte and (**B**) plasma. For a detailed description of lipid metabolism in white adipocyte see Sethi and Vidal-Puig (2007)^106^.

In the present study, we focused on the most significant relationships between pollutants and biomarkers of energy metabolism (i.e. highest ranked models with pollutants as predictors). Yet we cannot exclude the possibility that other pollutants not measured in this study could contribute to the disruption of energy metabolism in polar bears. For instance, suspect/non-target screening in polar bear tissue extracts indicated the presence of phthalates, nonylphenol and tonalide^27^, which are potential enhancers of adipogenesis^68,69^. Consequently, other pathways could also influence the relationships between energy metabolism and pollutants. For example, the RXR, the liver X receptor (LXR) in hepatocytes, and the pregnane X receptor (PXR) play a major role in the regulation of metabolism and are known targets for pollutants in mammals, including polar bears^70–72^. Furthermore, metabolic pathways can be interrupted or modified at several points and alter both expression and activity of proteins involved in lipid composition. For example, at the transcriptional level, *FASN* was positively related to ΣPCBs, whereas the FA *de novo* synthesis index in adipose tissue was negatively related to BDE-153. The relationship between FA *de novo* synthesis and BDE-153 could result from another pathway involving the “elongation of very long chain FA” (*ELOVL6*) gene^73^. The ELOVL6 enzyme controls FA composition in adipose tissue^74^. Consequently, BDE-153, which is positively related to FA elongation index could increase elongase activity, leading to increased polyunsaturated FAs, some of which can downregulate *de novo* lipogenesis^75,76^. Although it is not yet possible to establish a true causality chain, our data coupled to recent *in vitro* studies^27,72^ suggest that OHCs disrupt energy metabolism in free ranging female polar bears (Fig. 3). Studies on other marine mammals have also related transcript level changes in lipid metabolism to pollutant exposure^77,78^.

Although, the metabolome and the lipidome of female polar bears from Svalbard mainly clustered according to sampling season, we observed associations between the lipidome and pollutants. These results suggest that foraging ecology, which further controls body condition and pollutant exposure, is the main predictor of female polar bears’ metabolome and lipidome. In Northern elephant seals (*Mirounga angustirostris*), lactation and post-weaning fasting resulted in blood metabolome variation^79^. Moreover in female mink (*Neovison vison*), blood metabolome showed a clear clustering according to feeding regimen (restrictive vs. *ad libitum* feeding^80^). With regard to the assignment relationships between pollutants and the lipidome, these are tentative results, and should be followed up by detailed lipid characterization by MS/MS shotgun lipidomic methods^81,82^. Nevertheless, our results suggest that at the lipidome level, PCB, oxychlordane and BFRs could interact with the glycerophosphocholine pathway synthesis.

The consequences of associations between pollutants and parameters related to energy metabolism are for now unknown, but decreased *de novo* FA synthesis in polar bears with high pollutant exposure suggests that these individuals might be unable to convert excess carbohydrates to FAs. In addition, an excess of cholesterol synthesis related to PFAS may have subsequent effects on reproduction and health, since cholesterol is the precursor to all steroid hormones such as progesterone, testosterone, estradiol and glucocorticoids^83–85^. Consequently, a disruption of cholesterogenesis could influence much more than lipid metabolism and can be a starting point to steroid hormone disruption in polar bears^86^ and contribute to a number of metabolic diseases^87^.

In the current context of climate change, a reduction in sea ice extent may increase the energy that polar bears allocate to hunting (e.g. swimming longer distances or occupying larger home ranges); a finely tuned use of lipid stores is therefore required in an unpredictable environment. Combined effects of pollutants and climate change have been considered as a worst-case scenario to Arctic wildlife^88^. Here, we show that the response of energy metabolism to pollutants are more contrasted between more and less polluted females during periods with little sea ice^39^. Although sample size was low when splitting the dataset into eight classes, our results suggest that combining stressful environmental conditions to high concentrations of pollutants may act additively or synergistically to disrupt energy metabolism in polar bears. This study highlights the consequences of two anthropogenic threats to Arctic wildlife that need to be better understood and identified in order to preserve Arctic wildlife. Further studies are needed to reveal consequences of a disrupted energy metabolism in polar bears on fitness.

## Methods

### Field sampling

A detailed description of fieldwork, sample collection, storage, breeding status and geographical habitat categorization are given in the supporting information. Briefly, 112 adult female polar bears (estimated age 4–28 years) from the Barents Sea subpopulation were captured non-selectively in Svalbard during two seasons, spring (April) and autumn (September) 2012 and 2013. The bears were immobilized by remote injection and blood samples and adipose tissue biopsies were collected from each bear.

Fieldwork was carried out in accordance with relevant guidelines and regulations from the Governor of Svalbard and was approved by the Norwegian national animal research authority (FOTS).

### Gene description and transcript level analysis by relative quantitative real-time PCR (qPCR)

The studied genes in adipose tissue included *PPARG*, *PGC1, FASN, PNPLA2, LIPE, ADIPOQ, CD36, FABP4* and *SREBP1*. Total RNA was extracted from adipose tissue samples in the 112 polar bear samples using RNeasy Lipid Tissue Mini kit (QIAGEN, Oslo Norway) and cDNA produced from 1 μg RNA using qScript cDNA Synthesis Kit (Quanta Biosciences, Massachusetts, US). Quantitative real-time PCR was performed using SYBR Green I Master on a LightCycler® 480 (Roche, Basel, Switzerland). Detailed information on cycling conditions, establishment of standard curves, amplification efficiency and specificity is found in the supporting information (Table S2, Figure S1). Specific amplification of target and reference genes was confirmed by evaluation of melting curves and by agarose gel electrophoresis (Supporting information). Gene transcript levels were analyzed at the University of Bergen (UiB, Bergen, Norway).

### Determination of FAs from adipose tissue and FA indexes

Proportional mass data for 33 FAs were derived from 83 of the 112 polar bear samples, following methods described in the supporting information. We calculated two ratios between individual FAs as proxies of 1) FA *de novo* synthesis, which provides information on the conversion of excess carbohydrates to FAs and triacylglycerol^89^ and 2) FA elongation which provides information on the elongation of saturated and monounsaturated FA into very long chain FAs^90^. The ratios between individual FAs were calculated as follows: FA *de novo* synthesis (16:0/18:2n − 6)^89^ and FA elongation index ((18:0 + 18:1n − 9)/16:0)^73,90^. FA analysis was conducted at York University (Toronto, ON, Canada) and Dalhousie University (Halifax, NS, Canada).

### Lipid parameters determination in plasma

Plasma cholesterol, triglyceride and HDL concentrations (n = 111, mmol/L) were determined using a “dry” clinical-chemical analyzer, Reflotron® (Model IV, Boehringer-Mannheim GmhB, Mannheim, Germany) at the Norwegian University of Science and Technology (NTNU, Trondheim, Norway). Methods are detailed in the supporting information.

### Metabolome and lipidome

In plasma samples, polar metabolites (n = 111) and lipids (n = 101), were separated as detailed in the supporting information. The detected metabolite features, including ion source fragments, adducts and isotopic ions, are defined by a unique combination of m/z and retention time value^91^. The metabolome (low molecular weight polar metabolites) was characterized by nuclear magnetic resonance spectroscopy. The lipidome was characterized by fingerprints obtained from flow injection time-of-flight mass spectrometry with electrospray ionization. The most important polar metabolites that were affected by environmental and/or physiological factors were quantified by integration against trimethyl-silyl-propionate in MestreNova 8.1 (MestreLab Research S.L) and lipids were quantified by their exact mass (m/z) via Fourier transform ion cyclotron resonance. Metabolome and lipidome were measured at SINTEF (Trondheim, Norway).

### Pollutant determination in plasma and fat

OHCs were determined at the Norwegian University of Life Science (NMBU, Oslo, Norway). Concentrations of lipophilic pollutants (ng/g wet weight) such as PCBs, OCPs and BFRs were determined in adipose tissue (n = 111), whereas non-lipophilic pollutants such as perfluoroalkyl substances (PFASs) were determined in plasma (n = 112). Methods and limits of detection (LOD) have been reported elsewhere^39,92^. The compounds used for statistical analyses were those that were above the LOD in more than 90% of the samples. In adipose tissue, this was the case for: 14 PCBs (CB-99, -118, -137, -138, -153, -156, -157, -170, -180, -183, -189, -194, -206 and -209), oxychlordane, *trans*-nonachlor, HCB, and four BFRs (BDE-47, -100, -153, hexabromocyclododecane). In plasma, the concentrations of the following compounds were detected in >90% of the samples: six PFCAs with 8 to13 carbons (perfluorooctanoate, perfluorononanoate, perfluorodecanoate, perfluoroundecanoate, perfluorododecanoate and perfluorotridecanoate) and two PFSAs with 6 and 8 carbons (perfluorohexane sulfonate and perfluorooctane sulfonate). If a compound had one or more value below LOD we generated random numbers (“*runif”* function in the software R-3.2.5) ranging between the LOD value and ½ LOD. The pollutant levels and variations according to season, body condition diet and breeding status have already been discussed in previous studies^39,92^.

### Combined effects of pollutants and sea ice conditions

During the four sampling periods of this study (April 2012, September 2012, April 2013 and September 2013), April 2013 was considered as a “stressful period” due to the severely poor sea ice conditions during the previous winter^39^. During winter/spring 2013, Svalbard was mostly ice-free and sea ice concentrations rarely exceeded 12.5%, as opposed to during winter/spring 2012 when sea ice concentrations varied from 25% to more than 50%^93^. Furthermore, the abundance of preferred sea ice habitat available to polar bears was low during the months preceding the sampling period in April 2013 compared to the months preceding the other sampling periods^92^. As compared to the other 3 capture periods, in April 2013 a larger proportion of female polar bears were under nutritional stress as suggested by lower body condition, larger proportion of fasting females (higher urea to creatinine ratio) and higher *δ* ^15^N values, suggesting that females were catabolizing their protein pool, thus under nutritional stress^39,92^.

### Data availability and statistical analyses

All data are made available on the Norwegian Polar Institute data repository (data.npolar.no). Statistical analyses were performed using R 3-2.5^94^. First, we investigated the effects of metabolic state (i.e. if the bears were fasting versus feeding) on biomarkers of energy metabolism. To do so, we considered season as a proxy of metabolic state and assumed that most females captured in April were feeding whereas most females captured in September were fasting^92^. We then tested the effect of season on biomarkers of energy metabolism by using GLMM (R package *nlme* version 3.1–121^95^) with female identity as a random factor.

The best predictors of the concentrations of lipophilic and proteinophilic pollutants in the polar bears used in this study were previously determined to be body condition and diet, respectively, which are both related to season^31,39,92,96^. We tested the effects of age, body condition index (BCI), body mass and diet proxies (nitrogen and carbon stable isotope values in red blood cells) on energy metabolism biomarkers (Table S3). All energy biomarkers were either predicted by BCI, body mass or diet proxies (Table S3) and as previously mentioned BCI, body mass and diet proxies are all confounded with season^31,39,92,96^. To remove the possible confounding effect of season and test the effect of pollutants on metabolism biomarkers, we used GLMMs with pollutant concentrations as fixed factors, and, female identity and season as random factors. Prior to this, we performed a RDA^97,98^ to visualize the potential relationships between pollutants and biomarkers, and the pollutants were then grouped according to their proximity in the RDA loading plot. Then, for each response variable we built ten candidate models that included all the pollutants, individually or summed and the null model. We used conditional model averaging to make inferences from all the GLMMs. To rank the models we used an information-theoretic approach^99^ based on Akaike’s information criterion corrected for small sample size (AICc, R package *MuMIn*)^100^. The number of parameters (K), the difference in AICc values between the “best” model and the model at hand (ΔAICc) and a normalized weight of evidence in favor of the specific model, relative to the whole set of candidate models, derived by e^(−0.5(ΔAICc))^ (AICc weights) were calculated. We calculated averaged estimates for all predictor variables in the candidate model list, weighted using the AICc weights^99,101^. We obtained conditional parameter-averaged estimates (β) and 95% confidence intervals (CIs) for the predictors (individual or summed pollutants) included in the most competitive models (Table 1). We used 95% CI of the model averaged estimates to determine if parameters were significantly different from 0 at the 5% level. To determine if season could predict metabolome and lipidome raw data, we used PLS and MANOVA. Further, we studied relationships between the lipidome and pollutant concentrations. Using the mixOmics library in R^102^ a sparse PLS^103,104^ with multi-Y response was conducted with pollutant concentrations as response variables and the lipidome as predictor variables. Keeping 29 response and predictor variables, correlations between lipids and pollutants were observed.

Finally, to test for possible combined effects of sea ice conditions and internal pollutant levels on energy metabolism, we checked if the response of biomarkers of energy metabolism to pollutant concentrations differed between sampling periods qualified as “normal” such as April 2012, September 2012 and September 2013 or “stressful” (April 2013). We selected the pollutants that mostly influenced the biomarkers of energy metabolism (see Table 1). For each group of pollutant (best predictor according to AICc), the data set was split into two classes separated by the median pollutant concentration, resulting in one high and one low polluted group for each individual or group of pollutants. Due to variation in the internal concentrations of various pollutants in the bears, the individual bears were not necessarily the same in each group, according to the pollutant considered. Then, we compared the responses (concentrations, transcript levels) of biomarkers of energy metabolism between more and less polluted females for each period (spring 2012, autumn 2012, spring 2013 and autumn 2013). We used least square means (LSMs) comparisons to check whether the metabolic response of female polar bears from the more or less polluted groups differed for each sampling period. In unbalanced factorial experiments, LSMs for each factor mimic the main-effects means, but are adjusted for imbalance^105^.

## Electronic supplementary material

Supplementary information

## References

[1] Lee D-H, Porta M, Jacobs DR, Vandenberg LN (2014). Chlorinated Persistent Organic Pollutants, Obesity, and Type 2 Diabetes. Endocr. Rev..

[2] Ntambi JM, Young-Cheul K (2000). Adipocyte Differentiation and Gene Expression. J. Nutr..

[3] Ottaviani E, Malagoli D, Franceschi C (2011). The evolution of the adipose tissue: A neglected enigma. Gen. Comp. Endocrinol..

[4] Coelho M, Oliveira T, Fernandes R (2013). State of the art paper <br> Biochemistry of adipose tissue: an endocrine organ. Arch. Med. Sci..

[5] Vidal-Puig A (1996). Regulation of PPAR gamma gene expression by nutrition and obesity in rodents. J. Clin. Invest..

[6] Medina-Gomez G, Gray S, Vidal-Puig A (2007). Adipogenesis and lipotoxicity: role of peroxisome proliferator-activated receptor γ (PPARγ) and PPARγcoactivator-1 (PGC1). Public Health Nutr..

[7] Kim JB (1998). Nutritional and insulin regulation of fatty acid synthetase and leptin gene expression through ADD1/SREBP1. J. Clin. Invest..

[8] Janani C, Ranjitha Kumari BD (2015). PPAR gamma gene – A review. Diabetes Metab. Syndr. Clin. Res. Rev..

[9] Tontonoz P, Spiegelman BM (2008). Fat and Beyond: The Diverse Biology of PPARγ. Annu. Rev. Biochem..

[10] Desvergne B, Michalik L, Wahli W (2006). Transcriptional Regulation of Metabolism. Physiol. Rev..

[11] Sul H, Wang D (1998). Nutritional and hormonal regulation of enzymes in fat synthesis: Studies of Fatty Acid Synthase and Mitochondrial Glycerol-3-Phosphate Acyltransferase Gene Transcription. Annu. Rev. Nutr..

[12] Yeaman SJ, Smith GM, Jepson CA, Wood SL, Emmison N (1994). The multifunctional role of hormone-sensitive lipase in lipid metabolism. Adv. Enzyme Regul..

[13] Berg AH, Combs TP, Scherer PE (2002). ACRP30/adiponectin: an adipokine regulating glucose and lipid metabolism. Trends Endocrinol. Metab..

[14] Qiao L, Kinney B, Schaack J, Shao J (2011). Adiponectin Inhibits Lipolysis in Mouse Adipocytes. Diabetes.

[15] Grün F, Blumberg B (2009). Endocrine disrupters as obesogens. Mol. Cell. Endocrinol..

[16] Grün F, Blumberg B (2009). Minireview: The Case for Obesogens. Mol. Endocrinol..

[17] Gore AC (2015). EDC-2: The Endocrine Society’s Second Scientific Statement on Endocrine-Disrupting Chemicals. Endocr. Rev..

[18] Heindel JJ, Newbold R, Schug TT (2015). Endocrine disruptors and obesity. Nat. Rev. Endocrinol..

[19] Janesick AS, Blumberg B (2016). Obesogens: an emerging threat to public health. Am. J. Obstet. Gynecol..

[20] Letcher RJ (2010). Exposure and effects assessment of persistent organohalogen contaminants in arctic wildlife and fish. Sci. Total Environ..

[21] Blix AS (2016). Adaptations to polar life in mammals and birds. J. Exp. Biol..

[22] Liu S (2014). Population Genomics Reveal Recent Speciation and Rapid Evolutionary Adaptation in Polar Bears. Cell.

[23] Welch AJ (2014). Polar Bears Exhibit Genome-Wide Signatures of Bioenergetic Adaptation to Life in the ArcticEnvironment. Genome Biol. Evol..

[24] Atkinson, S. & Ramsay, M. The effects of prolonged fasting of the body composition and reproductive success of female polar bears (Ursus maritimus). *Funct. Ecol*. 559–567 (1995).

[25] Messier F, Taylor MK, Ramsay MA (1992). Seasonal activity patterns of female polar bears (Ursus maritimus) in the Canadian Arctic as revealed by satellite telemetry. J. Zool..

[26] Amstrup, S. C. Polar bear, Ursus maritimus. *Wild Mamm. N. Am. Biol. Manag. Conserv* (2003).

[27] Routti, H. *et al*. Environmental Chemicals Modulate Polar Bear (Ursus maritimus) Peroxisome Proliferator-Activated Receptor Gamma (PPARG) and Adipogenesis *in Vitro*. *Environ. Sci. Technol*. 10.1021/acs.est.6b03020 (2016). 10.1021/acs.est.6b03020 27602593

[28] Braathen M (2004). Relationships between PCBs and thyroid hormones and retinol in female and male polar bears. Environ. Health Perspect..

[29] Bytingsvik J (2013). Transthyretin-Binding Activity of Contaminants in Blood from Polar Bear (Ursus maritimus) Cubs. Environ. Sci. Technol..

[30] Villanger GD (2011). Exposure to mixtures of organohalogen contaminants and associative interactions with thyroid hormones in East Greenland polar bears (Ursus maritimus). Environ. Int..

[31] Bourgeon S (2017). Potentiation of ecological factors on the disruption of thyroid hormones by organo-halogenated contaminants in female polar bears (Ursus maritimus) from the Barents Sea. Environ. Res..

[32] Simon E (2013). Effect-Directed Analysis To Explore the Polar Bear Exposome: Identification of Thyroid Hormone Disrupting Compounds in Plasma. Environ. Sci. Technol..

[33] Pucci E, Chiovato L, Pinchera A (2000). Thyroid and lipid metabolism. Int. J. Obes..

[34] Knudsen N (2005). Small Differences in Thyroid Function May Be Important for Body Mass Index and the Occurrence of Obesity in the Population. J. Clin. Endocrinol. Metab..

[35] Jenssen BM (2015). Anthropogenic flank attack on polar bears: interacting consequences of climate warming and pollutant exposure. Front. Ecol. Evol..

[36] Derocher AE (2010). Climate change: The prospects for polar bears. Nature.

[37] Stirling I, Derocher AE (2012). Effects of climate warming on polar bears: a review of the evidence. Glob. Change Biol..

[38] Rode KD (2014). Variation in the response of an Arctic top predator experiencing habitat loss: feeding and reproductive ecology of two polar bear populations. Glob. Change Biol..

[39] Tartu S (2017). Sea ice-associated decline in body condition leads to increased concentrations of lipophilic pollutants in polar bears (Ursus maritimus) from Svalbard, Norway. Sci. Total Environ..

[40] Laidre KL (2015). Arctic marine mammal population status, sea ice habitat loss, and conservation recommendations for the 21st century. Conserv. Biol..

[41] Smithwick M (2005). Circumpolar Study of Perfluoroalkyl Contaminants in Polar Bears (Ursus maritimus). Environ. Sci. Technol..

[42] Verreault J (2005). Chlorinated hydrocarbon contaminants and metabolites in polar bears (Ursus maritimus) from Alaska, Canada, East Greenland, and Svalbard: 1996−2002. Sci. Total Environ..

[43] Muir DCG (2006). Brominated Flame Retardants in Polar Bears (Ursus maritimus) from Alaska, the Canadian Arctic, East Greenland, and Svalbard. Environ. Sci. Technol..

[44] McKinney MA (2011). Regional Contamination versus Regional Dietary Differences: Understanding Geographic Variation in Brominated and Chlorinated Contaminant Levels in Polar Bears. Environ. Sci. Technol..

[45] Dietz R (2015). Physiologically-based pharmacokinetic modelling of immune, reproductive and carcinogenic effects from contaminant exposure in polar bears (Ursus maritimus) across the Arctic. Environ. Res..

[46] Bando K (2010). Influences of biofluid sample collection and handling procedures on GC–MS based metabolomic studies. J. Biosci. Bioeng..

[47] Yin P, Lehmann R, Xu G (2015). Effects of pre-analytical processes on blood samples used in metabolomics studies. Anal. Bioanal. Chem..

[48] Cattet, M. Biochemical and physiological aspects of obesity, high fat diet, and prolonged fasting in free-ranging polar bears. (University of Saskatchewan, 2000).

[49] Yamauchi T (2002). Adiponectin stimulates glucose utilization and fatty-acid oxidation by activating AMP-activated protein kinase. Nat. Med..

[50] Berndt J (2007). Fatty acid synthase gene expression in human adipose tissue: association with obesity and type 2 diabetes. Diabetologia.

[51] Schulman IG, Shao G, Heyman RA (1998). Transactivation by Retinoid X Receptor–Peroxisome Proliferator-Activated Receptor γ (PPARγ) Heterodimers: Intermolecular Synergy Requires Only the PPARγ Hormone-Dependent Activation Function. Mol. Cell. Biol..

[52] Evans RM, Mangelsdorf DJ (2014). Nuclear Receptors, RXR, and the Big Bang. Cell.

[53] Coleman RA, Mashek DG (2011). Mammalian Triacylglycerol Metabolism: Synthesis, Lipolysis and Signaling. Chem. Rev..

[54] Park YM, Kashyap SR, Major JA, Silverstein RL (2012). Insulin promotes macrophage foam cell formation: potential implications in diabetes-related atherosclerosis. Lab. Invest..

[55] He J, Lee JH, Febbraio M, Xie W (2011). The emerging roles of fatty acid translocase/CD36 and the aryl hydrocarbon receptor in fatty liver disease. Exp. Biol. Med..

[56] Safe S (1993). Toxicology, Structure-Function Relationship, and Human and Environmental Health Impacts of Polychlorinated Biphenyls: Progress and Problems. Environ. Health Perspect..

[57] Lee JH (2010). A Novel Role for the Dioxin Receptor in Fatty Acid Metabolism and Hepatic Steatosis. Gastroenterology.

[58] Ogata M, Izushi F (1991). Effects of chlordane on parameters of liver and muscle toxicity in man and experimental animals. Toxicol. Lett..

[59] Bondy GS (2000). trans-Nonachlor and cis-Nonachlor Toxicity in Sprague-Dawley Rats: Comparison with Technical Chlordane. Toxicol. Sci..

[60] Ellero, S. L. *et al*. Xenobiotic-Metabolizing Cytochromes P450 in Human White Adipose Tissue: Expression and Induction. *Drug Metab. Dispos*. dmd.109.029249, 10.1124/dmd.109.029249 (2009). 10.1124/dmd.109.029249 20035023

[61] Duntas LH (2002). Thyroid disease and lipids. Thyroid Off. J. Am. Thyroid Assoc..

[62] Engelken SF, Eaton PR (1980). Thyroid Hormone-Induced Dissociation between Plasma Triglyceride and Cholesterol Regulation in the Rat. Endocrinology.

[63] Grover GJ (2004). Effects of the Thyroid Hormone Receptor Agonist GC-1 on Metabolic Rate and Cholesterol in Rats and Primates: Selective Actions Relative to 3,5,3′-Triiodo-l-Thyronine. Endocrinology.

[64] Viguerie N (2002). Regulation of Human Adipocyte Gene Expression by Thyroid Hormone. J. Clin. Endocrinol. Metab..

[65] Kim JB (1995). Dual DNA binding specificity of ADD1/SREBP1 controlled by a single amino acid in the basic helix-loop-helix domain. Mol. Cell. Biol..

[66] Foretz M, Guichard C, Ferré P, Foufelle F (1999). Sterol Regulatory Element Binding Protein-1c Is a Major Mediator of Insulin Action on the Hepatic Expression of Glucokinase and Lipogenesis-Related Genes. Proc. Natl. Acad. Sci. USA.

[67] Yoshizato K, Kikuyama S, Shioya N (1980). Stimulation of glucose utilization and lactate production in cultured human fibroblasts by thyroid hormone. Biochim. Biophys. Acta BBA - Gen. Subj..

[68] Pereira-Fernandes A (2013). Evaluation of a Screening System for Obesogenic Compounds: Screening of Endocrine Disrupting Compounds and Evaluation of the PPAR Dependency of the Effect. PLOS ONE.

[69] Hao C, Cheng X, Xia H, Ma X (2012). The endocrine disruptor 4-nonylphenol promotes adipocyte differentiation and induces obesity in mice. Cell. Physiol. Biochem..

[70] Francis GA, Fayard E, Picard F, Auwerx J (2003). Nuclear receptors and the control of metabolism. Annu. Rev. Physiol..

[71] Grün F, Blumberg B (2006). Environmental Obesogens: Organotins and Endocrine Disruption via Nuclear Receptor Signaling. Endocrinology.

[72] Lille-Langøy R (2015). Environmental contaminants activate human and polar bear (Ursus maritimus) pregnane X receptors (PXR, NR1I2) differently. Toxicol. Appl. Pharmacol..

[73] Green CD, Ozguden-Akkoc CG, Wang Y, Jump DB, Olson LK (2010). Role of fatty acid elongases in determination of de novo synthesized monounsaturated fatty acid species. J. Lipid Res..

[74] Shimano H (2012). Novel qualitative aspects of tissue fatty acids related to metabolic regulation: Lessons from Elovl6 knockout. Prog. Lipid Res..

[75] Wang Y (2004). The Human Fatty Acid Synthase Gene and De Novo Lipogenesis Are Coordinately Regulated in Human Adipose Tissue. J. Nutr..

[76] Catalá A (2013). Five Decades with Polyunsaturated Fatty Acids: Chemical Synthesis, Enzymatic Formation, Lipid Peroxidation and Its Biological Effects. J. Lipids J. Lipids.

[77] Castelli MG, Rusten M, Goksøyr A, Routti H (2014). mRNA expression of genes regulating lipid metabolism in ringed seals (Pusa hispida) from differently polluted areas. Aquat. Toxicol..

[78] Brown TM (2017). De novo assembly of the ringed seal (Pusa hispida) blubber transcriptome: A tool that enables identification of molecular health indicators associated with PCB exposure. Aquat. Toxicol..

[79] Champagne CD (2013). A profile of carbohydrate metabolites in the fasting northern elephant seal. Comp. Biochem. Physiol. Part D Genomics Proteomics.

[80] Hedemann MS, Damgaard BM (2012). Metabolomic study of plasma from female mink (Neovison vison) with low and high residual feed intake during restrictive and ad libitum feeding. Comp. Biochem. Physiol. Part D Genomics Proteomics.

[81] Han X, Gross RW (2005). Shotgun lipidomics: electrospray ionization mass spectrometric analysis and quantitation of cellular lipidomes directly from crude extracts of biological samples. Mass Spectrom. Rev..

[82] Bowden JA, Bangma JT, Kucklick JR (2014). Development of an automated multi-injection shotgun lipidomics approach using a triple quadrupole mass spectrometer. Lipids.

[83] Vos, J. G., Bossart, G., Fournier, M. & O’Shea, T. *Toxicology of Marine Mammals*. (CRC Press, 2003).

[84] Roberts KD, Bandi L, Calvin HI, Drucker WD, Lieberman S (1964). Evidence that Cholesterol Sulfate is a Precursor of Steroid Hormones. J. Am. Chem. Soc..

[85] Clayton RB (1965). Biosynthesis of sterols, steroids, and terpenoids. Part I. Biogenesis of cholesterol and the fundamental steps in terpenoid biosynthesis. Q. Rev. Chem. Soc..

[86] Pedersen KE, Letcher RJ, Sonne C, Dietz R, Styrishave B (2016). Per- and polyfluoroalkyl substances (PFASs) – New endocrine disruptors in polar bears (Ursus maritimus)?. Environ. Int..

[87] Maxfield FR, Tabas I (2005). Role of cholesterol and lipid organization in disease. Nature.

[88] Jenssen BM (2006). Endocrine-disrupting chemicals and climate change: a worst-case combination for arctic marine mammals and seabirds?. Environ. Health Perspect..

[89] Chong MF-F (2008). Parallel activation of de novo lipogenesis and stearoyl-CoA desaturase activity after 3 d of high-carbohydrate feeding. Am. J. Clin. Nutr..

[90] Jakobsson A, Westerberg R, Jacobsson A (2006). Fatty acid elongases in mammals: Their regulation and roles in metabolism. Prog. Lipid Res..

[91] Fei F, Bowdish DME, McCarry BE (2014). Comprehensive and simultaneous coverage of lipid and polar metabolites for endogenous cellular metabolomics using HILIC-TOF-MS. Anal. Bioanal. Chem..

[92] Tartu S (2017). Diet and metabolic state are the main factors determining concentrations of perfluoroalkyl substances in female polar bears from Svalbard. Environ. Pollut..

[93] Prop J (2015). Climate change and the increasing impact of polar bears on bird populations. Interdiscip. Clim. Stud..

[94] R Core Team. *R: A Language and Environment for Statistical Computing* (2016).

[95] Pinheiro, J., Bates, D., Debroy, S. & Sarkar, D. R core team. *nlme: Linear and Nonlinear Mixed Effects Model*s (2015).

[96] Tartu S (2016). Geographical Area and Life History Traits Influence Diet in an Arctic Marine Predator. PLOS ONE.

[97] Ramette A (2007). Multivariate analyses in microbial ecology. FEMS Microbiol. Ecol..

[98] Legendre P, Anderson MJ (1999). Distance-Based Redundancy Analysis: Testing Multispecies Responses in Multifactorial Ecological Experiments. Ecol. Monogr..

[99] *Model Selection and Multimodel Inference*. (Springer New York, 2004).

[100] Barton, K. *MuMIn: Multi-Model Inference* (2016).

[101] Lukacs PM, Burnham KP, Anderson DR (2009). Model selection bias and Freedman’s paradox. Ann. Inst. Stat. Math..

[102] Rohart, F., Gautier, B., Singh, A. & Cao, K.-A. L. mixOmics: an R package for’omics feature selection and multiple data integration. *bioRxiv* 108597, 10.1101/108597 (2017). 10.1371/journal.pcbi.1005752 PMC5687754 29099853

[103] Lê, C. K.-A., Rossouw, D., Robert-Granié, C. & Besse, P. A Sparse PLS for Variable Selection when Integrating Omics Data. *Stat. Appl. Genet. Mol. Biol*. **7** (2008). 10.2202/1544-6115.1390 19049491

[104] Lê Cao K-A, Martin PG, Robert-Granié C, Besse P (2009). Sparse canonical methods for biological data integration: application to a cross-platform study. BMC Bioinformatics.

[105] Lenth, R. V. & Hervé, M. lsmeans: Least-Squares Means (2015).

[106] Sethi JK, Vidal-Puig AJ (2007). Thematic review series: Adipocyte Biology. Adipose tissue function and plasticity orchestrate nutritional adaptation. J. Lipid Res..

