# Supplementary material for: Multiple-stressor effects in an apex predator: combined influence of pollutants and sea ice decline on lipid metabolism in polar bears
Source: Sci Rep. 2017 Nov 28;7:16487. doi: 10.1038/s41598-017-16820-5 (PMC5705648; doi:10.1038/s41598-017-16820-5)
Supplement: Supplementary file 1 — Supplementary information [file 41598_2017_16820_MOESM1_ESM.pdf]

SUPPORTING INFORMATION

Multiple-stressor effects in an apex predator: combined influence of pollutants and sea ice decline on lipid metabolism in polar bears

Sabrina Tartu[1*]; Roger Lille-Langøy[2]; Trond R. Størseth[3]; Sophie Bourgeon[1,4]; Anders Brunsvik[3;] Jon Aars[1]; Anders Goksøyr[2]; Bjørn Munro Jenssen[5]; Anuschka Polder[6]; Gregory W. Thiemann[7]; Vidar Torget[5] and Heli Routti[1]

[1] Norwegian Polar Institute, Fram Centre, NO-9296 Tromsø, Norway

[2] University of Bergen, Department of Biology, NO-5020 Bergen, Norway

[3] SINTEF, Trondheim, Norway

[4] UiT-The Arctic University of Norway, Department of Arctic and Marine Biology, NO- 9019 Tromsø, Norway

[5]Norwegian University of Science and Technology, Department of Biology, NO-7491 Trondheim, Norway

[6] Norwegian University of Life Science, Campus Adamstua, NO- 0033 Oslo, Norway

[7] York University, Faculty of Environmental Studies, Toronto, ON- M3J 1P3 Canada

*Corresponding author: Sabrina Tartu, Norwegian Polar Institute, Fram Centre, Postbox 6606 Langnes, 9296 Tromsø, Norway. Email: tartu.sabrina@gmail.com, phone: +47 77750538

Field sampling

Adult female polar bears (estimated age 4-28 years) from the Barents Sea subpopulation were captured non-selectively throughout Svalbard during two seasons, spring (April) and autumn (September) 2012 and 2013. The 112 samples collected represented 78 females. Twenty-six females were captured either twice (n=19), three times (n=6) or four times (n=1). Females were immobilized by remote injection of tiletamine hydrochloride and zolazepam hydrochloride (Zoletil Forte Vet ®; Virbac, France), delivered by a dart fired from a helicopter (Eurocopter AS350 Ecureuil). Blood samples were collected from the femoral vein using heparinized tubes and kept cool and out of sunlight until centrifuged within 10 h (3500 rpm, 9.65 cm rotor radius, 10 minutes). Adipose tissue samples were collected using an 8 mm biopsy punch, taken approximately 15 cm lateral to the base of the tail, the external portion of the biopsy was used for fatty acid analyses and the inner portion for transcript level analyses. Samples for transcript levels of genes and FA analyses were immediately placed in liquid nitrogen and stored at -80 ºC. Blood samples were kept cold and dark, and centrifuged within 10 hours (3500 rpm, 10 minutes). Plasma samples for the analyses of metabolome and lipidome were frozen in liquid nitrogen in the field and stored at -80ºC. Plasma and adipose tissue samples for the remaining analyses were frozen in the field and stored at -20 ºC until analyses. Immobilization and handling procedures followed standard protocols [1,2]. Females were classified in three groups according to their breeding status: solitary (i.e., alone, or in spring also occasionally together with a male), with one or two cubs of the year (COYs; cubs younger than one year old) or with one or two yearlings (cubs aged between 1 and 2 years). We divided Svalbard into two relative habitat categories with the western side considered as a poorer habitat and eastern side as a better habitat [3] according to a resource selection function [4]. Among the 78 individual bears used in this study, 59 were equipped with satellite telemetry collars during the study period or previous years. For these bears, we used location data to determine whether they used the good or poor habitats. For the bears without collars, we used the capture position of the individuals during the study period to determine if they were using good or poor habitats.

Transcription level analysis by quantitative PCR

Quantitative real-time PCR was performed using the LightCycler® 480 SYBR Green I Master on a LightCycler® 480 (Roche, Basel, Switzerland). Detailed information on primer sequences and cycling conditions can be found in Table S1. RefFinder was used to evaluate the expression stability of four candidate target reference genes, beta-actin (*ACTB*), glyceraldehyde-3-phosphate dehydrogenase (*GAPDH*), peptidylprolyl isomerase A (*PPIA*) and ribosomal protein L27 (*RPL27*). *ACTB* was identified as the most stable reference gene (results not shown) and transcript levels of nine target genes (**Table S1**) were normalized against expression level of *ACTB* (reference gene). Expression levels of target genes were expressed relative to the normalized expression level in a pooled sample.

Establishing standard curves and amplification efficiencies

Target and reference gene amplicons were subcloned in Strataclone pSC-A vectors (Agilent) that subsequently were linearized using Takara XbaI (Clontech, USA). Ten-fold serial dilution series of the linearized plasmids with seven different dilutions were used to establish a standard curve and to determine amplification efficiencies using the Second Derivative Maximum analysis method in the Roche LightCycler Software (version 1.5.0)

Evaluation of amplification specificity by melting peak analysis and agarose gel electrophoresis

Melting temperature analysis was performed after completed qPCR by briefly denaturing the products (95°C, 5 sec), allowing the products to anneal (65°C, 1 min) and finally heating the products to a maximum of 97°C with a ramp of 0.11C/sec. During the analysis fluorescence of the samples were measured 5 times/°C on a Roche LightCycler 480 instrument. Amplification products were also evaluated by agarose gel electrophoresis (2% agarose in 0.5XTris-Boric acid-EDTA buffer), **Figure S1**.

Fatty acid determination in adipose tissue

Data on composition of fatty acids (FA) in polar bear adipose tissue were derived from 83 samples (64 individuals) as in the remainder, fat biopsies were too small to determine FA composition. Lipid was quantitatively extracted from each adipose tissue sample according to [5] and FA methyl esters (FAME)

were prepared using H_2SO_4 as a catalyst [6]. Duplicate analyses and identification of FAME were performed using temperature-programmed gas–liquid chromatography according to [7] and [8,9]. Samples were analyzed on a Perkin Elmer Autosystem II Capillary gas chromatograph with a flame ionization detector fitted with a flexible fused silica column (30 m × 0.25 mm inner diameter) coated with 50% cyanopropyl polysiloxane (0.25-μm film thickness) (DB-23; Agilent Technologies, Palo Alto, California, USA). FA data are expressed as the mass percentage of total FA ± 1 standard error of the mean (SEM). Individual FA are referred to by the shorthand nomenclature of carbon-chain length:number of double bonds, and position of the first double bond relative to the terminal methyl group. We collected blood and adipose tissue from all the captured females. From 75 FA determined in fat samples, 33 FA represented more than 0.2% of total mass % FA: i-14:0, c14:0, 14:1n-5, c15:0, c16:0, 16:1n-11, 16:1n-9, 16:1n-7, 16:1n-5, i-17:0, 16:2n-4, c17:0, c18:0, 18:1n-11, 18:1n-9, 18:1n-7, 18:1n-5, 18:2n-6, 18:3n-4, 18:3n-3, 18:4n-3, 20:1n-11, 20:1n-9, 20:1n-7, 20:2n-6, 20:4n-6, 20:4n-3, 20:5n-3, 22:1n-11, 22:1n-9, 21:5n-3, 22:5n-3 and 22:6n-3.

Determination of cholesterol, triglyceride and HDL concentrations in plasma

Analyses of cholesterol, triglycerides and high-density lipoprotein (HDL) in plasma were performed at the Norwegian University of Science and Technology (NTNU) using a "dry" clinical-chemical analyzer, Reflotron® (Model IV, Boehringer-Mannheim GmhB, Mannheim, Germany). Plasma samples were thawed in darkness prior to analysis. A 32 µL pipette (Reflotron®) was used to transfer plasma to the test strips (Roche Diagnostics, Mannheim, Germany). Duplicates were analysed, and if large differences between duplicates were observed (>20%), analyses were performed in triplicates. The average of all the measurements for each sample was used for the statistical analysis. For QA/QC, after every three to five samples, a control Reflotron® Clean + Check strip (Roche Diagnostics, Mannheim, Germany) was used to ensure that the optical system measured values within the reference range. Values within the reference range confirmed that the apparatus did not drift throughout the analysis. Limit of detection (LOD) for the analytes were 2.59 mmol/L for cholesterol, 0.26 mmol/L for HDL and 0.80 mmol/L for triglycerides. All the HDL samples scored above the upper limit of detection and had to be diluted with

0.9% NaCl physiological saline solution. Trials were performed with several dilution curves to check for linearity and find optimal dilution range. A 1:1 dilution was applied, as it was found optimal.

Metabolome and lipidome in plasma

To separate lipids and polar metabolites, plasma samples (360 µl) were added to a solution of chloroform, methanol and water to achieve a final ratio of 2:2:1.8 of chloroform methanol and water (Milli-Q), respectively. This is the ratio of solvents used for the Bligh and Dyer [10] lipid extraction method, and results in a bi-phasic solution with a protein precipitate in the middle between a bottom chloroform phase and an upper methanol water phase. The phases were separated and analysed by nuclear magnetic resonance spectroscopy (NMR) and flow injection time-of-flight mass spectrometry (TOF-MS). For NMR spectroscopy, a 400µl aliquot of the methanol: water phase was evaporated in a vacuum centrifuge at 300K. The extract was resuspended in 200 µl of deuterium oxide (D_2O) containing 1mM trimethyl-silyl-propionate (TMSP) as an internal reference. The samples were analyzed on a Bruker DRU 600 spectrometer fitted with a Bruker Samplejet autosampler. 32 FIDs were averaged for each sample and a 1 Hz line broadening and zero filling was applied before Fourier transform. Glucose, lactate and citrate were quantified by integration against TMSP in MestreNova 8.1 (MestreLab Research S.L). The chloroform phase was analysed by TOF-MS flow injection analysis using an Agilent 1100 HPLC system to introduce samples into a Agilent 6220 TOF MS spectrometer using a mobile phase consisting of 95:5 Acetonitrile:MQ-water containing 4mM Sodium acetate. The injection volume was 1µl and the flow used was 0.5 ml/min. Data were recorded in both positive and negative mode. Mass spectra (50-1200 m/z) of the resulting single peak were exported to mzML format and imported into R using the mzR library, which was used to bin the data to one decimal before export of the complete dataset to a csv-file. Multivariate analysis was peformed in Matlab (2012a, Mathworks, Inc.) using the PLS-Toolbox (7.0.1) from Eigenvector Research, Inc. The 10-0.5 ppm region of the NMR spectra was exported from MestreNova and after removal of the water region an initial PCA was performed on mean centered and normalized (total area) data. A total number of ten samples were excluded based on their Hotelling T2 values and Q Residuals. These samples had variant levels of lipids and the 1.32 ppm (1.31-1.36 ppm) peak of lactate was removed due to variation in the chemical shifts of this peak. After this,

further PCA and PLS-DA models were made. The binned MS data were subjected to PCA and PLS-DA after mean centering. Breeding status (i.e. females with or without cubs) did not influence the metabolome and the lipidome (MANOVA, Pillai trace test, F<1.31, p< 0.254).

REFERENCES

1. Stirling, I., Spencer, C. & Andriashek, D. Immobilization of polar bears (Ursus maritimus) with Telazol® in the Canadian Arctic. *J. Wildl. Dis.* **25,** 159–168 (1989).
2. Derocher, A. E. & Wiig, Ø. Postnatal growth in body length and mass of polar bears (Ursus maritimus) at Svalbard. *J. Zool.* **256,** 343–349 (2002).
3. Tartu, S. *et al.* Diet and metabolic state are the main factors determining concentrations of perfluoroalkyl substances in female polar bears from Svalbard. *Environ. Pollut.* **229,** 146–158 (2017).
4. Lone, K., Merkel, B., Lydersen, C., Kovacs, K. M. & Aars, J. Sea ice resource selection models for polar bears in the Barents Sea subpopulation. *Ecography* n/a-n/a (2017). doi:10.1111/ecog.03020
5. Iverson, S. J., Lang, S. L. & Cooper, M. H. Comparison of the Bligh and Dyer and Folch methods for total lipid determination in a broad range of marine tissue. *Lipids* **36,** 1283–1287 (2001).
6. Thiemann, G. W., Budge, S. M. & Iverson, S. J. Determining blubber fatty acid composition: a comparison of in situ direct and traditional methods. *Mar. Mammal Sci.* **20,** 284–295 (2004).
7. Iverson, S. J., Frost, K. J. & Lang, L. Fat content and fatty acid composition of forage fish and invertebrates in Prince William Sound, Alaska: factors contributing to among and within species variability. *Mar. Ecol. Prog. Ser.* **241,** 161–181 (2002).
8. Budge, S. M., Iverson, S. J., Bowen, W. D. & Ackman, R. G. Among-and within-species variability in fatty acid signatures of marine fish and invertebrates on the Scotian Shelf, Georges Bank, and southern Gulf of St. Lawrence. *Can. J. Fish. Aquat. Sci.* **59,** 886–898 (2002).
9. Budge, S. M., Iverson, S. J. & Koopman, H. N. Studying Trophic Ecology in Marine Ecosystems Using Fatty Acids: A Primer on Analysis and Interpretation. *Mar. Mammal Sci.* **22,** 759–801 (2006).

10. Bligh, E. G. & Dyer, W. J. A Rapid Method of Total Lipid Extraction and Purification. *Can. J. Biochem. Physiol.* **37,** 911–917 (1959).

Table S1: Biomarkers of energy metabolism in relation to season (spring - feeding and autumn - fasting) in Svalbard female polar bears (2012-2013). Transcript levels are in arbitrary units and FA indexes were calculated as follows: FA *de novo* synthesis (16:0/18:2n-6) and FA elongation index ((18:0 + 18:1n-9)/16:0). Values are estimates and 95%CI obtained from mixed models using season as a predictor and female identity as a random factor. Values in bold are different from 0 at the 5% level.

energy metabolism biomarkers	Intercept and 95% CI (Spring)	Estimate and 95% CI
Adipose tissue		
PPARG	1.34 [1.14; 1.54]	**-0.42 [-0.71; -0.13]**
PGC1	1.38 [1.18; 1.59]	**-0.38 [-0.68; -0.08]**
FASN	1.52 [1.27; 1.76]	**-0.68 [-1.05; -0.31]**
PNPLA2	1.15 [0.98; 1.31]	-0.04 [-0.26; 0.19]
LIPE	0.97 [0.82; 1.12]	0.17 [-0.04; 0.37]
ADIPOQ	1.06 [0.84; 1.27]	0.10 [-0.18; 0.38]
CD36	1.04 [0.83; 1.26]	0.24 [-0.08; 0.55]
FABP4	1.02 [0.78; 1.25]	0.34 [0; 0.68]
SREBP1	1.07 [0.92; 1.21]	**0.33 [0.14; 0.52]**
**FA *de novo* synthesis index**	4.05 [3.72; 4.39]	**0.97 [0.5; 1.44]**
**FA elongation index**	4.04 [3.65; 4.44]	**-0.70 [-1.33; -0.07]**
Plasma		
Cholesterol (mmoL/L)	8.2 [7.77; 8.64]	0.38 [-0.22; 0.97]
HDL (mmoL/L)	2.86 [2.69; 3.02]	0.17 [-0.07; 0.42]
**Triglycerides (mmoL/L)**	1.45 [1.32; 1.58]	**0.5 [0.31; 0.69]**

Table S2: Target and reference genes in study, including details of primers used, estimated product sizes and amplification efficiencies. *****ACTB was most stably expressed (RefFinder) and used as reference gene for relative transcript level analysis. Thermo cycling was performed with a 10 second preincubation at 95°C followed by a 45 cycle amplification including a 10 seconds denaturation step at 95°C, a 10 second annealing step at a primer specific temperature and a 20 second elongation step at 72°C.

**Gene** (Name/*symbol*)	**Primer sequences** (5´→3´´)		**Tan (℃)**	**Product size** (bp)	**Efficiency**
Target genes/genes of interest (GOI)					
Fatty Acid Synthase	Fwd	CCTCTATCCGCTGGGTCT	60	96	1.94
(*FASN*)	Rev	GGAAGTTGAGAGAGGCATAGTT			
Sterol regulatory element binding transcription factor 1	Fwd	CACACCCAGGTCCAGAATG	60	94	1.72
(*SREBP1*)	Rev	GAGGTCACTGCGGTTGTT			
Peroxisome proliferator-activated receptor gamma, coactivator 1 alpha	Fwd	GCAGAAGGCAATTGAAGAGCG	57	223	1.86
(PGC1)	Rev	TTCGTTTGACCTGCGCAAAG			
Peroxisome proliferator-activated receptor gamma	Fwd	CACAATGGCCATCAGGTTTGG	57	281	1.9
(*PPARG*)	Rev	GGGGGTGATGTGTTTGAACT			
Adiponectin	Fwd	TGGATGAGAGTCCTGGGTATAG	60	95	1.76
(*ADIPOQ*)	Rev	CAAAGGGACTCAGAGGTGAAG			
Fatty acid binding protein 4	Fwd	GAAGTAGGAGTGGGCTTTGC	57	208	1.81
(FABP4/aP2)	Rev	AGGACACCTCCATCTAAGGTT			
Fatty acid translocase CD36	Fwd	CTCCTTGGCCTGGTAGAAATTA	57	87	1.92
(*CD36*)	Rev	TGATCTGCATGCACAGTATGA			
Patatin-like phospholipase domain Containing 2	Fwd	GGGTTGCCTGCTGAAGAT	60	121	1.86
(*PNPLA2*)	Rev	CTTGGAGCTGAAGTGGGATATG			
Heat sensitive lipase	Fwd	GTGAAGGACAGGACAGTGAGG	57	1.86	1.73
(*LIPE*)	Rev	CTCTTGAGGTAGGGCTCATG			
Candidate reference genes					
Glyceraldehyde-3-phosphate dehydrogenase	Fwd	ATCTTCCAGGAGCGAGATCC	57	250	1.86
(*GAPDH*)	Rev	GTGCTAAGCAGTTGGTGGTG			
Peptidylprolyl isomerase A	Fwd	GTCTCCTTTGAGCTGTTTGC	57	291	1.86
(*PPIA*)	Rev	AGTCTTGGCAGTGCAGATGA			
Actin, beta	Fwd	ACCCAGATCATGTTTGAGACC	57	229	1.89
(*ACTB*)*	Rev	TGATGTCACGCACGATTTCC			
Ribosomal protein L27	Fwd	ATGAAACCTGGGAAGGTGGT	57	237	1.88
(*RPL27*)	Rev	ACCTTGTGGGCATGAGGTGA			

Table S3: Relationships between biomarkers of energy metabolism and age, body condition index, body mass, diet proxies (*$\delta^{13}C$* and *$\delta^{15}N$*) and season in female polar bears adipose tissue and plasma captured in Svalbard (2012-2013). Values are ΔAICc, parameter estimates and 95% confidence intervals derived from conditional model averaging of general linear mixed models that included female identity as a random factor. Values in bold are the most competitive models including the best predictor (ΔAICc=0).

	Age		BCI		Body mass		*$\delta^{13}C$*		*$\delta^{15}N$*		Season		Null model
	Δ AICc	β[95 % CI]	Δ AICc	β[95 % CI]	Δ AICc	β[95 % CI]	Δ AICc	β[95 % CI]	Δ AICc	β[95 % CI]	Δ AICc	β[95 % CI]	Δ AICc
Transcript levels													
PPARG	7.4		8.33		7.89		4.74		2.97	0.15 [0.02; 0.28]	**0**	**-0.3 [-0.51; -0.09]**	6.36
PGC1	7.28		7.27		5.82		5.73		**0**	**0.18 [0.05; 0.31]**	**0.93**	**-0.27 [-0.48; -0.06]**	5.17
FASN	12.8		12.17		11.7		9.84		8.2	0.17 [0.01; 0.33]	**0**	**-0.48 [-0.74; -0.21]**	10.73
PNPLA2	7.67		5.42		6.88		**0.38**	**0.24 [0.07; 0.41]**	**0**	**0.14 [0.04; 0.25]**	7.73		5.7
LIPE	7.24		**0**	**0.22 [0.07; 0.37]**	3.28		**0.51**	**0.22 [0.07; 0.38]**	**0.36**	**0.14 [0.04; 0.23]**	5.53		6.05
ADIPOQ	7.66		**0**	**0.3 [0.1; 0.49]**	4.4		4.61	0.23 [0.02; 0.45]	4.64	0.14 [0.01; 0.28]	8.48		6.95
CD36	7.43		**1.22**	**0.29 [0.07; 0.52]**	4.4		**0**	**0.33 [0.1; 0.56]**	**1.95**	**0.17 [0.03; 0.31]**	5.85		5.89
FABP4	11.6		**0**	**0.46 [0.22; 0.7]**	4.49		6.66	0.34 [0.09; 0.59]	9.72		9.88		11.53
SREBP1	9.95		5.12	0.17 [0.01; 0.33]	9.41		**0**	**0.26 [0.1; 0.42]**	4.37	0.12 [0.02; 0.22]	**0.09**	**0.23 [0.09; 0.36]**	7.82
FA indexes													
de novo synthesis	20.8		**0**	**0.92 [0.52; 1.31]**	8.5	0.02 [0.01; 0.03]	20.23		11.74	-0.37 [-0.62; -0.12]	6.17	0.69 [0.35; 1.02]	18.81
Elongation	32		**0**	**-1.23 [-1.65; -0.81]**	11.7	-0.02 [-0.03; -0.01]	32		29.43		26.97	-0.50 [-0.95; -0.04]	29.87
Plasma parameters													
Cholesterol	5.83		2.64		**0**	**-0.01 [-0.023; -0.003]**	6.13		5.78		5.21		4.59
HDL	5.8		5.06		**0.97**	**-0.004 [-0.088; -0.0004]**	2.45		**0**	**0.13 [0.02; 0.23]**	3.84		3.67
Triglycerides	24.7		20.49		24.7		23.79		24.16		**0**	**0.35 [0.22; 0.49]**	22.57
Glucose	6.74	0.07 [0.01; 0.13]	12.07		10.7		11.89		12.04		**0**	**-0.78 [-1.22; -0.33]**	9.92

Lactate	7.69	5.83	3.46	9.85	9.33	**0**	**-0.07 [-0.12; -0.03]**	7.7

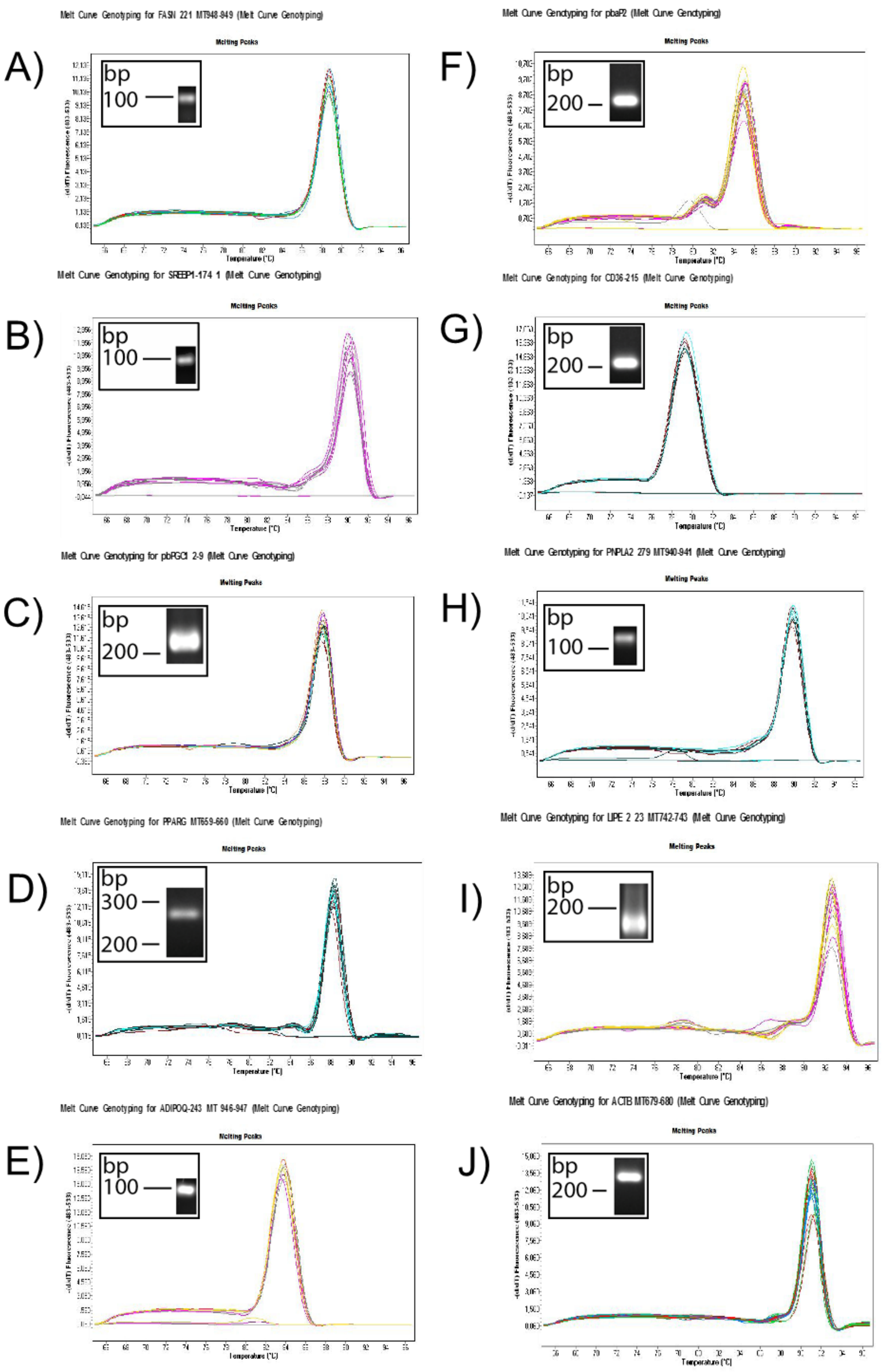

Figure S1 - Evaluation of amplification product specificity. Melting peaks and amplification products in a 2% agarose gel (0.5XTBE) for A) *FASN*, B) *SREBP1*, C) *PGC1*, D) *PPARG*, E) *ADIPOQ*, F) *FABP4/aP2*, G) *CD36*, H) *PNPLA2*, I) *LIPE* J) *ACTB*.

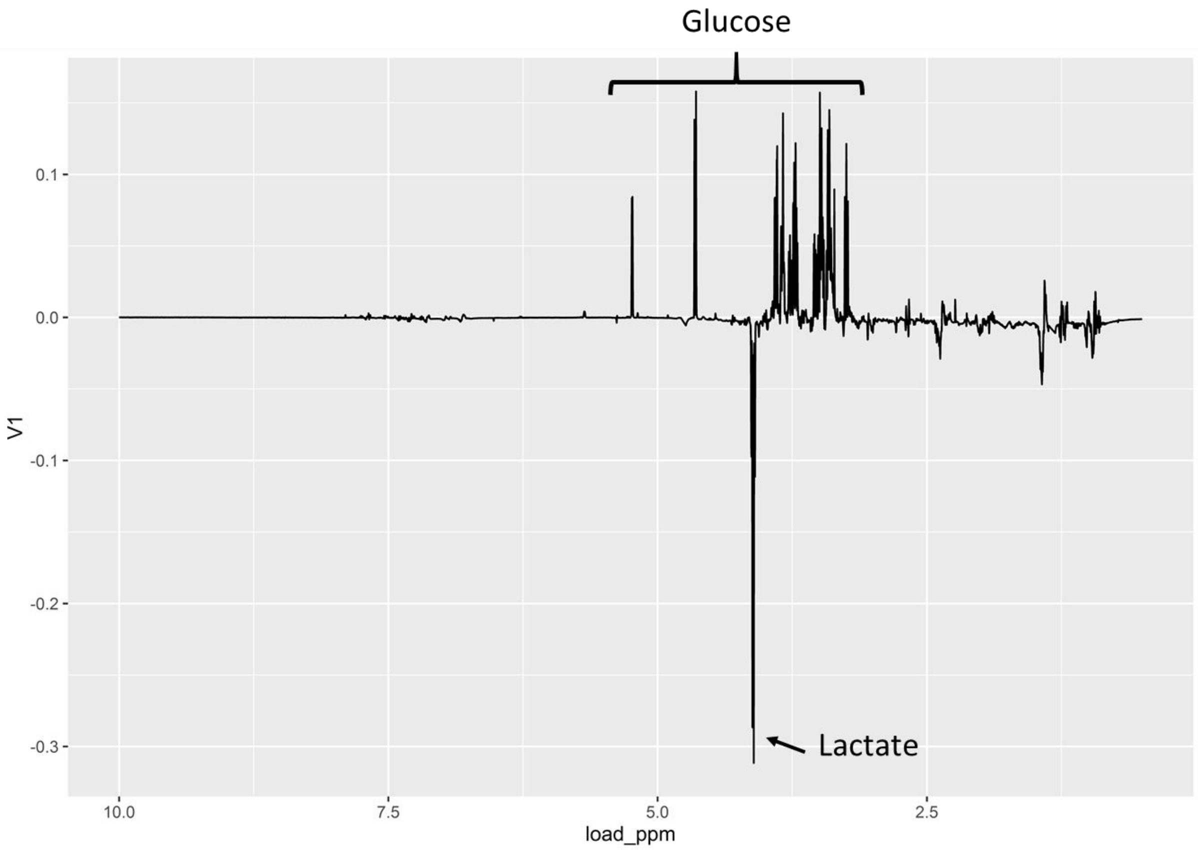

Figure S2: Metabolome loadings in plasma from female polar bears captured in Svalbard (2012-2013).
